# Eomesodermin-expressing T-helper cells are essential for chronic neuroinflammation

**DOI:** 10.1038/ncomms9437

**Published:** 2015-10-05

**Authors:** Ben J. E. Raveney, Shinji Oki, Hirohiko Hohjoh, Masakazu Nakamura, Wakiro Sato, Miho Murata, Takashi Yamamura

**Affiliations:** 1 Department of Immunology, National Institute of Neuroscience, National Center of Neurology and Psychiatry, 4-1-1 Ogawa-Higashi,Kodaira, Tokyo 187-8502, Japan; 2 Department of Molecular Pharmacology, National Institute of Neuroscience, National Center of Neurology and Psychiatry, Tokyo 187-8502, Japan; 3 Multiple Sclerosis Center, National Center Hospital, National Center of Neurology and Psychiatry, Tokyo 187-8502, Japan; 4 Department of Neurology, National Center Hospital, National Center of Neurology and Psychiatry, Tokyo 187-8502, Japan

## Abstract

Development of acute experimental autoimmune encephalomyelitis (EAE) depends on Th17 cells expressing the nuclear factor NR4A2. However, in mice lacking NR4A2 in T cells, a late-onset disease is still inducible, despite a great reduction in acute inflammation. We here reveal that development of this late onset disease depends on cytotoxic T-cell-like CD4^+^ T cells expressing the T-box transcription factor Eomesodermin (Eomes). T-cell-specific deletion of the *Eomes* gene remarkably ameliorates the late-onset EAE. Strikingly, similar Eomes^+^ CD4^+^ T cells are increased in the peripheral blood and cerebrospinal fluid from patients in a progressive state of multiple sclerosis. Collective data indicate an involvement of granzyme B and protease-activated receptor-1 in the neuroinflammation mediated by Eomes^+^ CD4^+^ T cells.

## Abstract (web summary)

Eomesodermin is a master regulator of effector CD8+ T cells. Here the authors show that it also plays a critical role in pathogenic CD4+ cells in a mouse model of multiple sclerosis, and its inactivation ameliorates the chronic stage of the disease.

## Graphical abstract

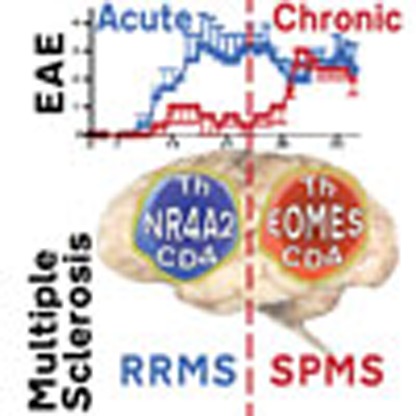

Recent research relying on genome-wide association studies^1^,^2^,^3^ has successfully identified a number of genes significantly linked with the pathogenesis of autoimmune diseases such as multiple sclerosis (MS). In the case of MS, the vast majority of the susceptibility genes have key roles in the functions of T helper (Th) cells and cellular immune responses^3^. These results support the relevance of research towards clarifying the development, differentiation and functions of Th cells, to identify new targets of therapy for autoimmune diseases.

NR4A2, also known as Nurr1, is an orphan nuclear receptor that is upregulated in CD4^+^ T cells derived from patients with the relapsing-remitting form of MS (RRMS)^4^,^5^. NR4A2 upregulation was also observed in CD4^+^ T cells infiltrating the central nervous system (CNS) and in peripheral blood of mice with experimental autoimmune encephalomyelitis (EAE), an animal model of MS^4^,^6^. This transcription factor was first described as an immediate/early response gene necessary for the development of neurons and their excitatory activity^7^,^8^,^9^. However, its role as an early response gene in CD4^+^ T-cell activation^6^, including Foxp3^+^ regulatory T cells^10^, has been recently demonstrated.

We have previously revealed that NR4A2 plays a critical role in the production of interleukin (IL)-21 and IL-17 from Th17 cells^6^. Consistently, small interfering RNA (siRNA)-induced inhibition of NR4A2 expression ameliorated the signs of EAE, showing that Th17 cell-mediated acute inflammation in EAE is under the control of NR4A2. To further establish the role of NR4A2 in autoimmune inflammation, we generated conditional knockout (cKO) mice whose expression of NR4A2 is deleted under the control of CD4 expression in all T cells. As expected, the new NR4A2 cKO mice developed only very mild signs of early/acute EAE. However, to our great surprise, clinical signs of EAE in the mice worsened rapidly around 3–4 weeks after sensitization, reaching equivalent levels to those in the control mice, and persisted over months thereafter. We postulated that the late/chronic stage of this EAE model does not require NR4A2-dependent Th17 cells, although NR4A2-expressing CD4^+^ T cells do play a major role in the early/acute phase. These results prompted us to examine the differences between early/acute and late/chronic inflammation in EAE. Subsequently, we found that inflammatory CD4^+^ T cells in the CNS during late/chronic EAE strikingly upregulated the T-box transcription factor Eomesodermin (Eomes)^11^,^12^. Studies using Eomes KO mice and *Eomes*-specific siRNA knockdown revealed that this transcription factor is critically involved in the development of the late/chronic stage of EAE induced in NR4A2 cKO mice.

MS is an autoimmune disease driven by invasion of autoimmune Th1 and Th17 cells into the CNS^13^,^14^. An inflammatory cascade triggered by entry of such pathogenic T cells promotes demyelination and axonal changes characteristic of MS lesions^15^,^16^,^17^. In the early stage, most patients have RRMS with recurrent disabling neurological symptoms. A majority of RRMS patients later develop a chronic progressive disease without signs of relapses, known as secondary progressive MS (SPMS)^18^,^19^,^20^. Reflecting the limited understanding of the pathogenesis, treatment and control of SPMS are challenging for both patients and physicians. Given the homology in the clinical picture between SPMS and late/chronic EAE, we have explored whether or not Eomes^+^ CD4^+^ T cells might be also involved in the pathogenesis of SPMS. Consistently, we found that Eomes^+^CD4^+^ T cells were remarkably increased in the peripheral blood and cerebrospinal fluid (CSF) of SPMS patients, compared with healthy subjects and patients with RRMS. Eomes^+^CD4^+^ T cells derived from rodent EAE and human MS were found to share some properties of cytotoxic CD8^+^ T cells, such as granzyme B expression and degranulation on activation as revealed by surface CD107a expression.

It has recently been described that granzyme B could mediate perforin-independent neurotoxicity caused by activated T cells^21^. In this model of neurotoxicity, granzyme B binding to protease-activated receptor (PAR)-1 expressed on neuronal cells triggers neurodegeneration, following an increased expression of the potassium channel Kv1.3 and activation of Notch-1. Surface expression of Kv1.3 on neuronal cells is not detected under a physiological condition, but could be detected in cortical neurons adjacent to the active inflammatory lesions of MS^21^. To test whether or not Eomes^+^ CD4^+^ T cells could induce chronic neuroinflammation via a similar mechanism dependent on granzyme B and PAR-1, we treated the late/chronic phase of EAE with granzyme B-specific siRNA or with PAR-1 antagonists. The results showed that blockade of granzyme B and PAR-1 were both effective for reducing the signs of late/chronic EAE. As such, identification of Eomes^+^ CD4^+^ T cells as pathogenic lymphocytes in chronic CNS inflammation has a major implication in understanding the chronic CNS inflammation and designing a novel effective therapy for SPMS.

## Results

### EAE consists of NR4A2-dependent and -independent phases

**Figure 1:**
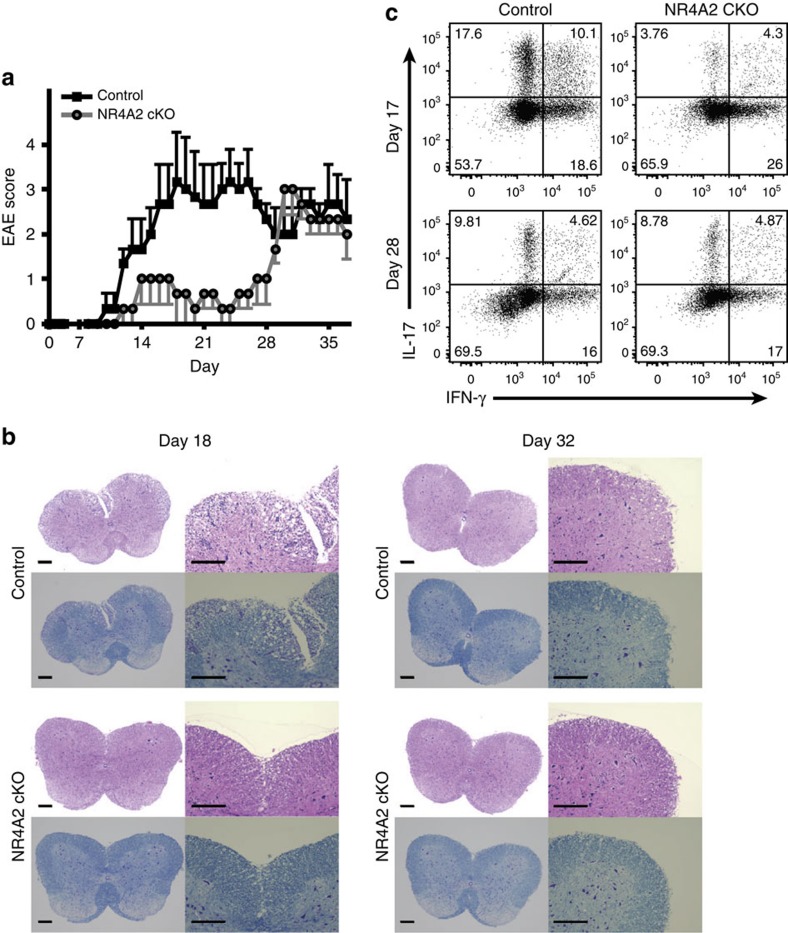
Mice lacking NR4A2 in CD4^+^ T cells are protected from early/acute EAE but develop late/chronic EAE signs. (**a**) Clinical EAE score. Disease scoring for control NR4A2^fl/fl^ (Control, black squares) and Cre-CD4/NR4A2^fl/fl^ (NR4A2 cKO, grey circles). The mice with C57BL/6 background were immunized with MOG_35–55_ peptide emulsified in complete Freund’s adjuvant. Error bars represent s.e.m. (**b**) Histopathology of EAE. The spinal cords were fixed in formal-saline, paraffin embedded and sectioned before microphotography. Panels show adjacent sections from a representative mouse stained with haematoxylin and eosin (top row) or Luxol fast blue (bottom row) at × 4 (left) or × 10 (right) magnification; top panels are from a control mouse and bottom panels are from a NR4A2 cKO mouse; left panels are from day 18 post EAE induction, right panels are from day 32 post EAE induction from 1 of 2 independent experiments. Scale bars, 200 μm. (**c**) IL-17 and IFN-γ production by CNS CD4^+^ T cells as measured by intracellular cytokine staining following PMA/ionomycin stimulation in the presence of GolgiPlug for 5 h. The cells were isolated by enzymatic digestion of pooled CNS tissues during either early (Day 17) or late phase (Day 28) of EAE. Data are representative of at least three independent experiments, each using pools of three to five mice for each genotype and time point.

As siRNA blockade of NR4A2 expression led to a great reduction of IL-17 production from Th17 cells^4^,^6^, we set out to further investigate the biological significance of NR4A2 in autoimmune inflammation. We generated a cKO mouse on the B6 background with a CD4-specific deletion of *nr4a2* (NR4A2 cKO). When these mice and control mice were immunized with MOG_35–55_ peptide to induce EAE (Fig. 1a), NR4A2 cKO mice showed a significantly delayed EAE onset and had very low clinical severity during the early/acute phase as compared with NR4A2 replete B6 mice (Control). This is consistent with the postulate that NR4A2 expressed by Th17 cells plays a critical role in initiating the early/acute phase of EAE. Surprisingly, around a month after immunization, clinical signs of NR4A2 cKO mice rapidly increased. Afterwards, both Control and NR4A2 cKO mice had a similar course of EAE with equivalent disease severity. Pathological evaluation (Fig. 1b) revealed a reduced cellular infiltration in NR4A2 cKO versus Control mice during early/acute phase EAE, but not during late/chronic phase, consistent with the results of clinical scoring. Flow cytometric analyses for intracellular IL-17 and interferon (IFN)-γ also demonstrated that numbers of Th17 cells infiltrated into the CNS are greatly reduced in NR4A2 cKO compared with control B6 mice during the early/acute phase of EAE (Day 17) (Fig. 1c), although the difference was not evident during chronic phase. Moreover, *ex vivo* cytokine production from the isolated CNS lymphocytes was consistent with the flow cytometery data (Supplementary Fig. 1A,B). The reduction of early/acute phase in the cKO mice was as expected, given the role of NR4A2 in pathogenic functions of Th17 cells^6^. However, preservation of the late/chronic phase was surprising, because suppression of acute inflammation is generally thought to prevent subsequent occurrence of chronic inflammation. Taken together, we propose that clinical stages of MOG_35–55_-induced EAE can be separated into two phases: an NR4A2-dependent early/acute phase and an NR4A2-independent late/chronic phase.

### *Eomes expression by CD4* ^*+*^ *T cells in late/chronic EAE*

**Figure 2:**
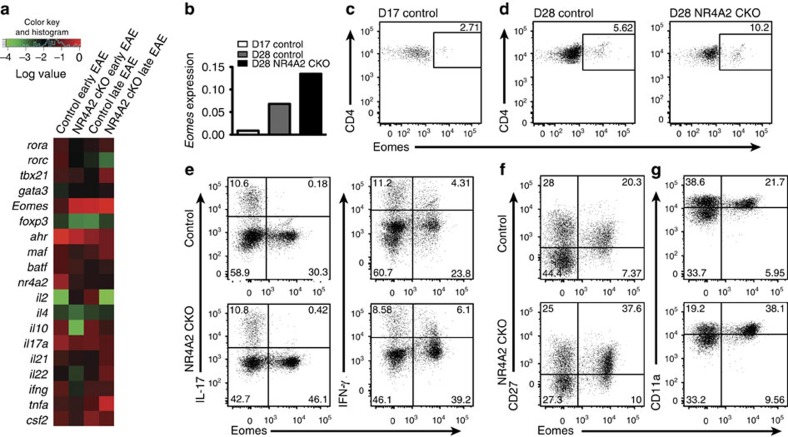
Late/chronic EAE is associated with *Eomes* expression by pathogenic CNS-infiltrating CD4^+^ T cells. (**a)** Differential expression of immune-associated genes. CD4^+^ T cells were isolated and sorted from control and NR4A2 cKO CNS from mice with early or late EAE, and analysed by expression microarray. Gene expression was confirmed by quantitative reverse transcriptase–PCR (qRT–PCR) and relative expressions of selected genes are summarized by heat map. (**b**) Differential expression of *eomes* in the CNS CD4^+^ T cells determined by qRT-PCR. (**c**,**d**) Intracellular flow cytometry for Eomes expression of freshly isolated CNS T cells. Unstimulated cells were stained intracellularly. (**e**–**g**) Flow cytometric analyses for co-expression of IL-17, IFN-γ, CD27 and CD11a, with Eomes in CD4^+^ CNS-infiltrating T cells (Day 27). The cells were stained after stimulation with PMA/ionomycin in the presence of GolgiPlug for 5 h. FACS staining plots are representative of at least three independent EAE experiments.

To elucidate potential pathogenic mechanisms operating in the late/chronic phase of EAE, we analysed gene expression profiles of CNS-infiltrating CD4^+^ T cells. DNA microarray analysis followed by real-time PCR confirmation has revealed a number of immune-related genes that are differentially expressed during early/acute and late/chronic phases (Fig. 2a,b). This analysis highlighted the expression of the *Eomes* gene (*Tbr2*) by CNS-infiltrating CD4^+^ T cells as being upregulated during the late/chronic phase of EAE in control and NR4A2 cKO mice. Although it has been broadly appreciated that cytotoxic CD8^+^ T cells and natural killer cells express this transcription factor^11^,^12^, *Eomes* expression by CD4^+^ T cells in the context of autoimmune inflammation has not been previously described to our knowledge. Flow cytometric analysis further revealed that proportions of CD4^+^ T cells expressing the Eomes protein are increased in chronic EAE lesions (Day 28) as compared with acute lesions (Day 17) (Fig. 2c,d). Signs of chronic EAE in both Control and NR4A2 cKO mice were persistent over months; analysis of CNS lymphocytes 15 weeks after immunization has revealed that Eomes^+^ CD4^+^ T cells, including those producing IFN-γ, are present in the chronic lesions (Supplementary Fig. 2). Although it was not emphasized in previous works, small numbers of Eomes^+^ CD4^+^ T cells are present in the lymphoid tissues from naïve mice (Supplementary Fig. 3A,B). We were curious to know whether Eomes^+^ CD4^+^ T cells actually resembled either Th1 or Th17 cells that play a pathogenic role in EAE. As previously described, pathogenic Th17 cells did not or only marginally expressed the CD27 costimulatory molecule, which mediates repression of Th17 cell functions via interaction with CD70 (ref. 22). However, Eomes^+^CD4^+^ T cells in the EAE lesions brightly expressed CD27. In accordance with this, they did not elaborate IL-17, although a proportion of the cells produced IFN-γ (Fig. 2e,f). Pathogenic Th17, but not Th1 cells, express CD11a, which is an adhesion molecule also expressed by tissue-resident memory CD4^+^ T cells^23^,^24^. The Eomes^+^CD4^+^ T cells expressed higher levels of CD11a than Eomes^−^ cells (Fig. 2g).

### *Eomes* ^*+*^ *CD4* ^*+*^ *T cells are required for late/chronic EAE*

**Figure 3:**
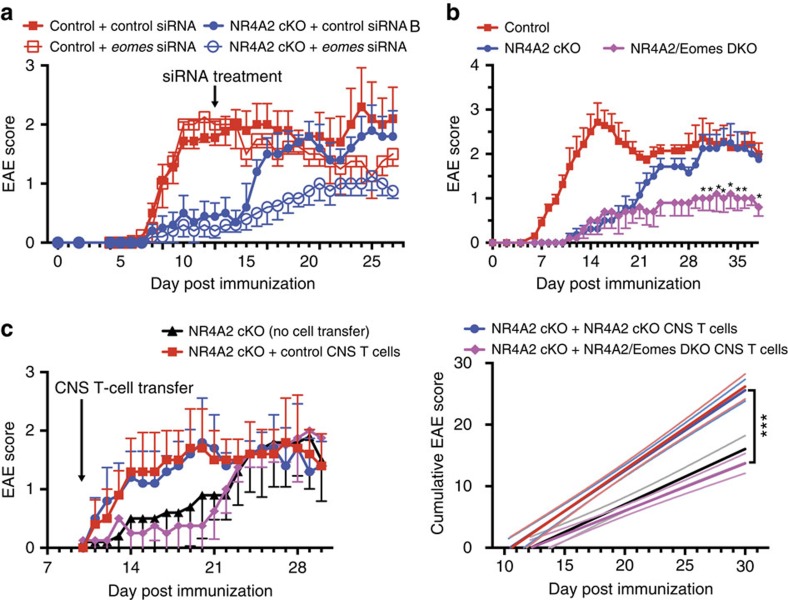
Role of Eomes expressed by CD4^+^ T cells in chronic CNS inflammation. (**a**) siRNA treatment for late/chronic EAE. *eomes*-specific (unfilled symbols) or control scrambled (solid symbols) siRNA was stabilized in an atellocollagen substrate and injected i.v. into control (red) or NR4A2 cKO mice (blue) on day 15 post EAE induction, to generate systemic gene knockdown. Plots show EAE scores for mice. Error bars represent s.e.m. for groups of five mice. (**b**) Induction of EAE in NR4A2/Eomes DKO mice compared with NR4A2 cKO mice. Plot shows clinical EAE scores for control (red symbols), NR4A2 cKO (blue symbols) and Eomes/NR4A2 dKO (magenta symbols) mice immunized with MOG_35–55_. Error bars represent s.e.m. for groups of five mice. (**c**) Exacerbation of EAE after transfer of CNS T cells. CD45^+^TCR^+^CD4^+^CD11b^−^F4/80^−^ FACS-sorted CNS T cells were isolated from control, NR4A2 cKO or NR4A2/Eomes DKO mice with late-stage EAE on day 28 post EAE induction. T cells were restimulated polyclonally with anti-CD3 monoclonal antibody for 48 h before adoptive transfer into NR4A2 cKO recipient mice that had been immunized with MOG_35–55_ 7 days before. Plots show clinical scores (left) and cumulative disease scores (right), and are representative of two independent experiments. Error bars represent s.e.m. for clinical scores for groups of four or five mice and light lines show the 95% confidence interval for cumulative disease scores.

To evaluate the role of Eomes during late/chronic EAE, we applied RNA interference to specifically block *Eomes* expression *in vivo*. Systemic administration of *Eomes*-specific siRNA after the peak of acute EAE (Day 15) significantly suppressed the severity of late/chronic EAE in NR4A2 cKO mice (Fig. 3a), as compared with administration of control siRNA. This siRNA treatment also reduced the clinical signs of late/chronic EAE in control B6 mice, albeit with a much reduced efficacy. These results suggested that Eomes upregulation might play a role in the formation of chronic CNS inflammation. Using mice with an *Eomes* deletion in T cells (under control of *CD4-cre*, termed Eomes cKO), we next tested whether or not *Eomes* expression by CD4^+^ T cells is required for mediating late/chronic EAE. The Eomes cKO mice immunized with MOG_35–55_ showed reduced EAE symptoms during the late/chronic stage, although only a marginal reduction was observed during early/acute EAE (Supplementary Fig. 4). We further generated double KO (DKO) mice, lacking both Eomes and NR4A2 in T cells (NR4A2/Eomes DKO), to assess the role of Eomes in the absence of NR4A2. In the DKO mice, signs of early/acute EAE did not differ from those in NR4A2 cKO mice. Strikingly, we found that late/chronic stage of EAE was markedly reduced in the DKO mice compared with NR4A2 cKO (Fig. 3b). This reduction in severity of EAE in the absence of Eomes was not a consequence of enhanced development of regulatory T cells expressing Foxp3 or producing IL-10 (Supplementary Fig. 5A,B). Taken together, we propose that the development of late/chronic EAE is promoted by pathogenic Eomes^+^CD4^+^ T cells infiltrating the CNS.

### *Eomes* ^*+*^ *T cells augment CNS inflammation on transfer*

As Eomes is reported to control IFN-γ production and cytotoxic function in CD8^+^ T cells^11^,^12^, and our KO system deleted *Eomes* from both CD4^+^ and CD8^+^ T cells, it was possible that a reduced production of IFN-γ from CD8^+^ T cells might account for the reduction in late/chronic disease in NR4A2/Eomes DKO mice. Therefore, we examined the IFN-γ-producing capacity of CNS-infiltrating T cells (Supplementary Fig. 5C,D). In the late/chronic phase of EAE in wild-type mice, the overall proportion of IFN-γ-producing CD8^+^ T cells in the CNS was much lower (<5%) than IFN-γ-producing CD4^+^ T cells. Moreover, despite similar clinical severity, CNS lymphocytes from late/chronic disease in NR4A2 cKO mice contained much higher proportions of IFN-γ-producing CD8^+^ T cells than Control mice (Supplementary Fig. 5C,D). These results were not consistent with the hypothesis that CNS-infiltrated CD8^+^ T cells were involved in the pathogenesis of late/chronic EAE symptoms in an Eomes-dependent manner. Thus, we decided to shift our focus towards investigating the pathogenic potential of Eomes^+^CD4^+^ T cells in the late EAE lesions. Accordingly, we isolated CD4^+^ CNS T cells from control or NR4A2 cKO mice with late/chronic EAE and transferred them into NR4A2 cKO mice that had just started to develop EAE. Although the signs of EAE in control NR4A2 cKO mice remained very mild (clinical score<1.0) over the following week, transfer of as few as 50,000 activated cells from either of the donor genotypes induced a surprisingly rapid worsening of EAE in the recipient mice (Fig. 3c). These T cells from late/chronic CNS lesions appear to require *Eomes* expression to exert the striking pathogenic activity, as similar transfer of CNS CD4^+^ T cells from NR4A2/Eomes DKO mice did not alter the course of EAE in NR4A2 cKO mice (Fig. 3c). We next transferred donor CNS CD4^+^ T cells after separating them into CD27^+^ and CD27^−^ fraction; as Th17 cells obligatory lack CD27, whereas Eomes^+^CD4^+^ T cells all express CD27, this separation enables rough separation of these cell types. Transfer of as few as 30,000 of CD27^+^CD4^+^ T cells from either genotypes provoked worsening of acute EAE in a couple of days (Supplementary Fig. 6A). EAE signs in control NR4A2 cKO mice reached the levels of the mice transferred with CNS CD27^+^CD4^+^ T cells more than 3 weeks later. Mice transferred with CD27^−^CD4^+^ CNS T cells from wild-type mice also induced an exacerbation of EAE, but there was an interval of 1 week between transfer and worsening. In contrast, CD27^−^CD4^+^ CNS T cells from NR4A2 cKO mice had no significant effects on EAE (Supplementary Fig. 6B). These results indicate that the pathogenic CD27^−^CD4^+^ T cells in the late/chronic disease might be NR4A2-expressing Th17 cells. Taken together, we postulated that the highly pathogenic CD27^+^CD4^+^ T cells capable of augmenting signs of EAE corresponded to Eomes^+^CD4^+^ T cells.

### *Cytotoxic properties associated with Eomes* ^*+*^ *T cells*

**Figure 4:**
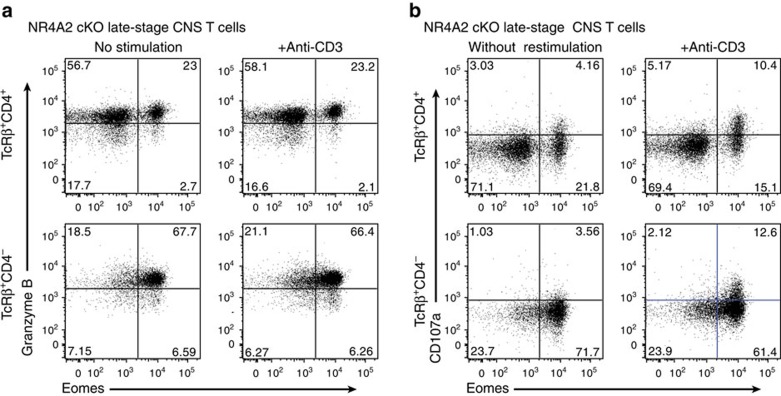
Eomes^+^CD4^+^ T cells mediate cytotoxicity. Pooled CNS T cells were isolated from NR4A2 cKO mice with late-stage EAE. Purified CD45^+^TcRβ^+^CD4^+^ cells and CD45^+^TcRβ^+^CD4^−^ cells were prepared by FACS sorting. These T cells were restimulated with or without anti-CD3 antibodies in the presence of CNS-infiltrating myeloid cells and fluorescently conjugated anti-CD107a antibodies. After 4 h stimulation, cells were then stained intracellularly for expression of Eomes and granzyme B. Plots show granzyme B expression (**a**) and CD107a surface trapping (**b**) for unstimulated (left) and restimulated (right) CNS CD4^+^ T cells (top) and CNS CD4^−^ T cells (bottom). Data are representative of three independent experiments.

Unlike pathogenic Th17 cells, Eomes^+^CD4^+^ T cells did not elaborate IL-17 and do not appear to require NR4A2 for their functional maturation. Although the pathogenic functions of Eomes^+^CD4^+^ T cells in autoimmunity have not been previously analysed, expression of Eomes is linked with cytotoxic function in CD8^+^ T cells and natural killer cells. More recently, it has been demonstrated that CD4^+^ Th cells can be reprogrammed to also act as cytotoxic effector T cells by repressing ThPOK, the key transcription factor that stabilizes CD4 lineage^25^. The results of these studies guided us to examine whether or not pathogenic Eomes^+^CD4^+^ T cells express molecules required for exerting cytotoxicity. We observed an increase in the cytotoxic protease granzyme B in CD4^+^ T cells from late/chronic EAE lesions in NR4A2 cKO mice (Fig. 4a). Furthermore, we found that Eomes^+^CD4^+^ T cells from late CNS lesions in NR4A2 cKO underwent lysosome degranulation on stimulation as evidenced by CD107a membrane fusion (Fig. 4b), suggesting that these cells could release lytic granules on activation.

### *Eomes* ^*+*^ *CD4* ^*+*^ *T cells in human MS*

**Figure 5:**
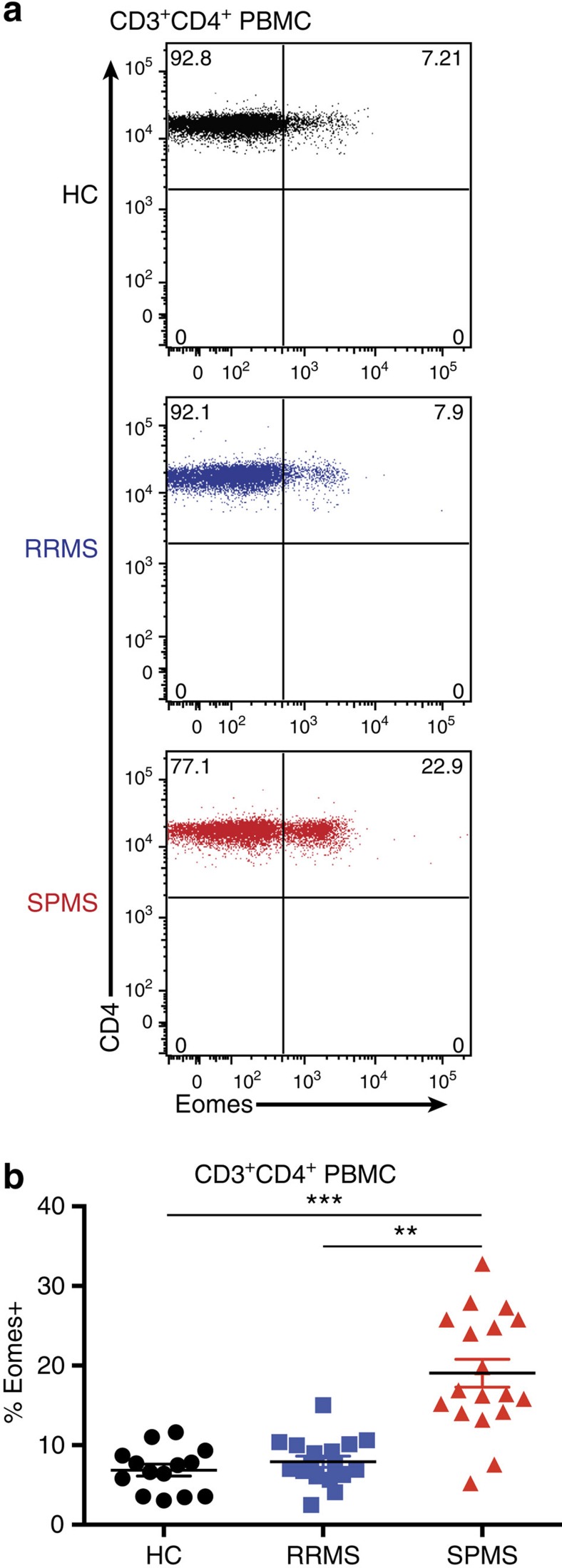
Increased proportions of Eomes^+^CD4^+^ T cells in patients with SPMS. (**a**) Eomes expression by peripheral blood T cells from HCs (plotted black), patients with RRMS (plotted blue) and patients with SPMS (plotted red) as measured by flow cytometry. (**b**) Proportions of Eomes^+^ cells (%) among CD3^+^CD4^+^ PBMCs. Horizontal lines shows mean % for each patient group with s.e.m. error bars. Sample group information is detailed in Supplementary Table 1. ****P<0.0001 and ***P<0.001 as measured by a two-tailed *T*-test.

**Figure 6:**
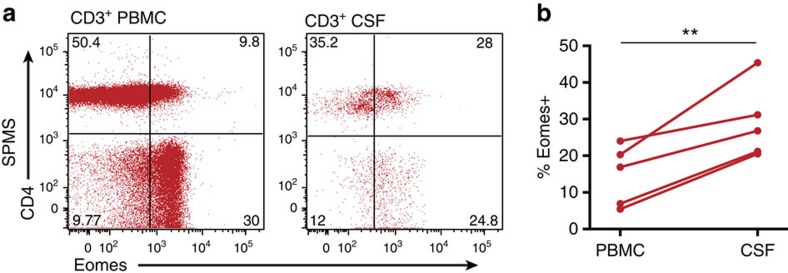
Accumulation of Eomes^+^CD4^+^ T cells in the CSF. **a**) Eomes expression by CD3^+^ T cells in peripheral blood or CSF samples from patients with SPMS. (**b**) Proportions (%) of Eomes^+^ cells among CD3^+^CD4^+^ from PBMCs and CSF. Results from each patient are connected with lines **P<0.01 with a Wilcoxon matched-pairs signed rank test.

**Figure 7:**
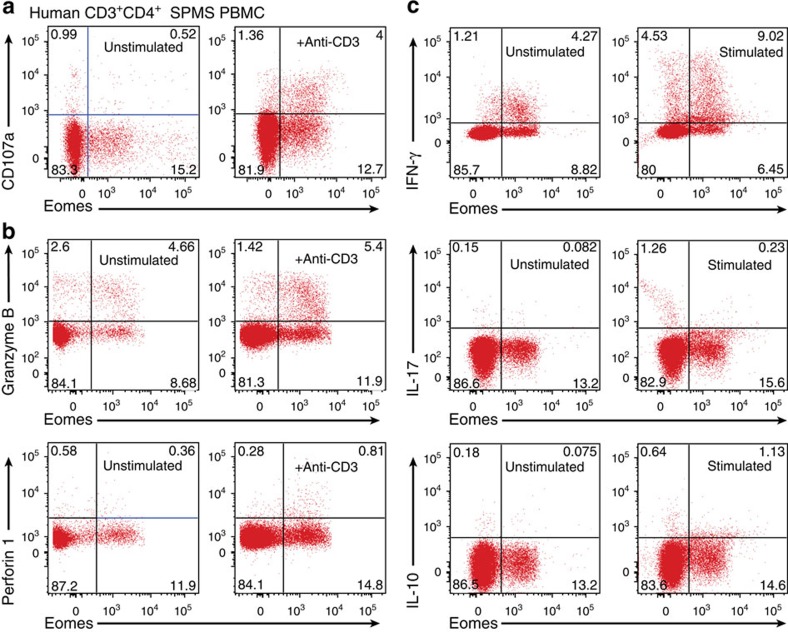
Eomes^+^CD4^+^ T cells from SPMS patients have characteristics of cytotoxic cells. (**a**) PBMCs from SPMS patients were cultured with or without anti-CD3 and anti-CD28 antibodies in the presence of fluorescently conjugated anti-CD107a antibodies and brefaldin A. After 5 h stimulation, cells were then stained intracellularly for expression of Eomes. Plots show CD107a surface trapping for unstimulated (left) and restimulated (right) for CD3^+^CD4^+^ T cells and granzyme B, and perforin 1 staining shown in **b**. (**c**) PBMCs from SPMS patients were stimulated with PMA/ionomycin in the presence of GolgiPlug. After 5 h stimulation, cells were stained intracellularly with antibodies against granzyme B, IFN-γ, IL-17 and IL-10. Plots show CD3^+^CD4^+^ PBMCs from a representative SPMS patient from four SPMS patients tested.

To evaluate whether or not the studies of the late/chronic EAE have implications for human MS, we examined *Eomes* expression by CD4^+^ T cells in peripheral blood mononuclear cells (PBMCs) derived from healthy controls (HCs), or patients with either RRMS or SPMS. Eomes^+^CD4^+^ T cells constituted 2%–12% of total CD4^+^ T cells in the PBMCs from HCs (Fig. 5), consistent with a recent report on human Eomes^+^CD4^+^ T cells in healthy individuals^26^. We observed that proportions (%) of Eomes^+^CD4^+^ T cells among CD4^+^ T cells in RRMS were not significantly different from HCs. However, the % of Eomes^+^ T cells was remarkably increased in patients with SPMS, ranging from 5% to 35%, indicating the striking homology between SPMS and late/chronic EAE. As observed in Eomes^+^CD4^+^ T cells in mice, a proportion of the cells expressed CD11a, and most Eomes^+^CD4^+^ T cells from HCs and RRMS were CD27^+^ (Supplementary Fig. 7A–D). Further, the increase of Eomes^+^ cells in SPMS was CD4^+^ T-cell-specific and was not observed in CD4^−^ T cells (Supplementary Fig. 7E). In addition, *Eomes* expression did not correlate with age, gender, immunomodulatory treatment, or current disability scores in individual patients with SPMS (Supplementary Fig. 7F–H). Previous works showed an association of *Eomes* expression with memory T-cell compartments and their functions^26^,^27^. We also observed an association of activated/memory phenotype (CD45RO^+^CD45RA^−^) with Eomes^+^CD4^+^ T cells (Supplementary Fig. 8). Moreover, proportions of Eomes^+^CD4^+^ T cells were further enriched in the CSF from patients with SPMS as compared with corresponding blood samples (Fig. 6a,b), indicating their propensity for moving to the site of autoimmune inflammation. Regarding the pathogenic functions, we confirmed that human Eomes^+^CD4^+^ T cells from SPMS also exhibited CD107a mobilization on stimulation (Fig. 7a) and expressed cytotoxic markers granzyme B and IFN-γ (Fig. 7b,c). These results indicate that Eomes^+^CD4^+^ T cells may also play a key pathogenic role in SPMS.

### Blocking Granzyme B/PAR-1 ameliorates late/chronic EAE

**Figure 8:**
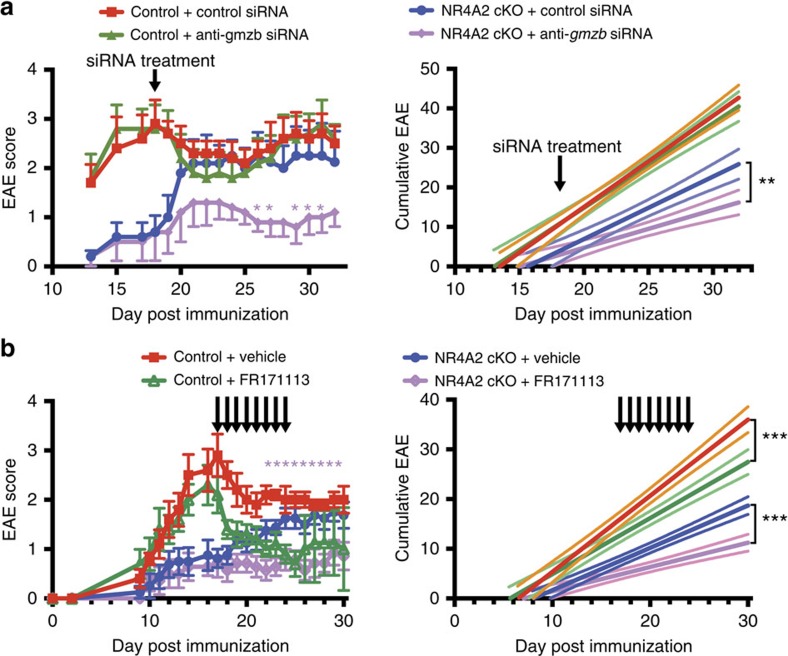
Effective treatment for late/chronic neuroinflammation. (**a**) Effects of granzyme B-specific siRNA for late/chronic EAE. NR4A2 cKO mice were immunized with MOG_35–55_ and five of the ten mice were treated with granzyme B-specific siRNA (anti-*gmzb* siRNA) stabilized in atellocollagen on day 17 post EAE induction (NR4A2 cKO+anti-grzb siRNA). As control, WT mice were immunized at the same day (control) (*n*=5 for each group). In the left panel, clinical EAE scores are shown with error bars representing s.e.m. *P<0.05 for treated versus untreated NR4A2 cKO mice as tested by a Fisher’s least significant difference (LSD) test. In the right panel, solid lines represent cumulative disease scores, with dashed lines showing 95% confidence intervals. ***P<0.001 tested by linear regression analysis. Data are representative of two independent experiments. (**b**) Effects of PAR antagonist FR171113 for late/chronic EAE. Groups of wild-type (WT) mice (control) or NR4A2 cKO mice were immunized with MOG_35–55_. One group of each genotype was treated daily from day 17 post EAE induction by intraperitoneal injection of PAR antagonist FR171113 or carboxymethyl cellulose (CMC) as vehicle. Left panel shows clinical EAE scores with error bars representing s.e.m. *P<0.05 for treated NR4A2 cKO versus untreated NR4A2 cKO mice as tested by a Fisher’s LSD test. Right panel shows cumulative disease, with dashed lines showing 95% confidence intervals. ***P<0.001 tested by linear regression analysis. *n*⩾5 and data are representative of three independent experiments.

CD4^+^ memory T cells and CD8^+^ T cells have been reported as mediating cytotolytic effects via granzyme B release^23^,^28^. When we administered siRNA specific for the gene encoding granzyme B, *gmzb*, late/chronic EAE induced in NR4A2 cKO was significantly suppressed (Fig. 8a). As pathogenic NR4A2-dependent Th17 cells, which are able to produce granzyme B^29^, are not present in the late chronic lesions, we postulated that Eomes^+^CD4^+^ T cells could be the major source of granzyme B that promotes development of chronic EAE. Cytotoxic mechanisms using granzymes usually requires co-release of perforin 1 that helps the entry of granzyme into target cells^30^, yet perforin 1 expression was hardly detected in human Eomes^+^CD4^+^ T cells. Curiously, in a recently described mechanism of perforin-independent cytotoxicity against neuronal cell culture^21^, granzyme B acts directly on PAR-1 receptor expressed by neurons, leading to activation of intracellular protease pathways inducing apoptosis. PAR-1 is one of four family members of serine protease activated G protein-coupled receptor. To test whether or not such a mechanism as found *in vitro* could be involved in the pathogenesis of late/chronic EAE mediated by Eomes^+^CD4^+^ T cells, we blocked the activation of PAR-1 receptor *in vivo*, using a PAR-1 antagonist, FR171113 (ref. 31). When we started the treatment at the peak of EAE (Day 17), subsequent development of late/chronic EAE and cumulative disease burden were significantly suppressed in both wild-type mice and NR4A2 cKO mice (Fig. 8b). Similar results were obtained when mice were instead treated with SCH79797 dihydrochloride, a selective inhibitor of PAR-1 (Supplementary Fig. 9). As such, not only blockade of Eomes, but also granzyme B and PAR-1 receptor, led to the amelioration of late/chronic EAE. Given that perforin-independent neuronal death can be mediated by granzyme B/PAR-1 interactions, our results raise the possibility that granzyme B-releasing Eomes^+^CD4^+^ T cells may use such a pathway to cause chronic inflammation in the CNS.

## Discussion

To test a new concept of autoimmunity or to deduce the pathogenesis of autoimmune diseases, EAE models have previously proven to be most useful^15^,^16^,^17^. However, the pathogenesis of chronic CNS inflammation leading to persistent or progressive neurological dysfunctions has not been fully analysed. Here we show that NR4A2 cKO mice immunized with MOG_35–55_ develop late-onset chronic EAE, despite greatly reduced signs of early inflammation. We propose that although early/acute EAE is under control of NR4A2-dependent Th17 cells, the late/chronic EAE is instead dependent on the T-box transcription factor Eomes^11^,^12^, mediated by T cells expressing *Eomes*. Critically, signs of acute and late EAE were greatly inhibited, when expression of both *Nr4a2* and *Eomes* genes were knocked down in T cells (Fig. 3b), indicating a reciprocal involvement of NR4A2 and Eomes in acute and late phases of EAE. The EAE induced in NR4A2 cKO mice allowed us to study the mechanism of chronic CNS inflammation without interference from concomitant acute inflammation mediated by Th17 cells. These mouse EAE studies have implications for human disease as Eomes^+^CD4^+^ T cells resembling those associated with late/chronic EAE were increased in the peripheral blood and CSF from patients with SPMS. The predominance of the Eomes^+^CD4^+^ T cells among the CSF lymphocytes further supported their role in the development of SPMS. A large genome-wide association study has demonstrated a significant link between *Eomes* gene polymorphism and predisposition to MS^32^, providing further support to the translational potential of our results.

*Eomes* is expressed in a proportion of CD4^+^ T cells both in naïve mice (Supplementary Fig. 3) and in healthy human subjects (Fig. 5). However, Eomes has been regarded as a transcription factor predominantly expressed by CD8^+^ T cells, which mediates IFN-γ production and arms T cells with cytotoxic properties^11^,^12^. Reminiscent of this, Eomes^+^CD4^+^ T cells from both EAE lesions and blood of SPMS have potentials to upregulate CD107a and release granzyme B and IFN-γ (Fig. 4). Besides autoimmunity, recent studies have revealed that Eomes^+^CD4^+^ T cells are generated under chronic inflammatory conditions associated with infection or can be induced in tumour microenvironments by immunomodulation^33^,^34^. Mechanistically, persistent T-cell stimulation via strong costimulation through tumour necrosis factor receptor family members, such as CD137 (4-1BB) signals^33^ or a combination of CD134 and CD137 signals^35^, has been shown to induce the Eomes^+^CD4^+^ T cells. Notably, critical roles of CD134L-CD134 and CD137L-CD137 interactions have been demonstrated in EAE^36^,^37^, and an increased level of CD137L was detected in MS patients’ peripheral blood^38^, implying that the costimulatory ligands associated with Eomes induction in CD4^+^ T cells are present in the CNS lesions of EAE and MS.

By extrapolating previous works^39^, transforming growth factor-β-mediated suppression of Eomes is a prerequisite for Th17 cell differentiation, suggesting a mutually exclusive expression of IL-17 and Eomes in Th cells. This would account for the absence of IL-17 production among Eomes^+^CD4^+^ T cells. Instead, Th cells may upregulate expression of Eomes after receiving continuous costimulatory signals concomitant with chronic inflammation *in vivo*^33^,^35^. The *in-vivo* origin of Eomes^+^CD4^+^ T cells is still controversial, as they show a mixed phenotype of Th1 cells and cytotoxic T lymphocytes, producing IFN-γ, expressing CD11a and exerting cytotoxicity. Alterations in ThPOK, the transcription factor that stabilizes CD4 expression and subdues cytotoxic pathways via the silencing of CD8^+^ T-cell differentiation factor Runx3, provide a possible pathway that may lead to *Eomes* expression in Th cells. Reduced expression of *ThPOK* has been shown to reprogramme Th cells to express *Eomes*, granzyme B, perforin and cytotoxic T lymphocyte functions^40^,^41^,^42^,^43^; therefore, marginal downmodulation of ThPOK under chronic inflammatory condition might lead to the acquired expression of *Eomes* in Th cells. It is noteworthy to point out that persistent expression of *Eomes* itself is not necessarily enough to confer pathogenicity, as Th cells isolated from NR4A2 cKO mice at early/acute stage of EAE showed a higher Eomes expression comparable with those observed in CNS-Th cells at later stage of EAE (Fig. 2a). It is likely to be that a further antigenic or even nonspecific activation is required to generate the degranulation, leading to pathogenic effects by these Eomes^+^ Th cells. As Eomes is deleted from both CD4^+^ and CD8^+^ T cells in our KO system, it was conceivable that CD8^+^ T cells could play a pathogenic role in late/chronic disease in an Eomes-dependent manner. Increased target organ-infiltrating CD8^+^ T cells have been observed during late/chronic disease in another model of organ-specific autoimmunity; however, these CD8^+^ T cells showed an exhausted phenotype and were not thought to contribute to persistent inflammation^44^. Indeed, we observed that proportions of IFN-γ-producing CD8^+^ T cells in the late CNS lesions were much lower than expected and our transfer experiments implicated CD4^+^ T cells in late/chronic CNS lesions as highly pathogenic in an Eomes-dependent manner. Thus, although we have not ruled out a disease-modulating property of Eomes^+^CD8^+^ T cells, our data indicated that Eomes^+^ CD4^+^ T cells played a pathogenic role in the late/chronic EAE.

The specific increase of Eomes^+^CD4^+^ T cells in SPMS was unexpected, as the role of T cells in SPMS has not always been highlighted in the past. A limited efficacy of immunomodulatory drugs in SPMS was rather interpreted as an active involvement of innate immune cells^18^. On the other hand, pathological studies emphasized the presence of secondary lymphoid follicles associated with SPMS^20^. Although we do not negate the role of innate immune cells or germinal centre B cells in SPMS, our results clearly point to a key role for cytotoxic CD4^+^ T cells in chronic neuroinflammation and thus direct future research direction towards analysis of Eomes^+^CD4^+^ T cells. In fact, the possible involvement of cytotoxic Eomes^+^CD4^+^ T cells in SPMS hints at a potential reason why current immunomodulatory therapies aimed at conventional pathogenic cells proved ineffective for SPMS^45^. Our proposal that such Eomes^+^ cells are directly cytotoxic to neuronal cells via granzyme B binding and degradation of PAR-1 receptors is supported by our preclinical studies. Thus, we suggest that targeting Eomes^+^CD4^+^ T cells and/or PAR-1 function may yield specific treatments for SPMS. Finally, the accumulation of Eomes^+^CD4^+^ T cells may prove to be a critical milestone in the development of RRMS into SPMS^46^.

## Methods

### Animals and EAE induction

All mice used were aged 6–8 weeks and were maintained in specific pathogen-free conditions in accordance with institutional guidelines. This study was approved by the Committee for Small Animal Research and Animal Welfare (National Center of Neurology and Psychiatry). All efforts were made to minimize animal suffering in clinical disease experiments where five to ten mice were used for scoring in each group. For generation of homozygous floxed *nr4a2* mice (*nr4a2*^*fl/fl*^), briefly the NR4A2 transgene flanked by loxp sites (Supplementary Fig. 10) was microinjected into C57/BL/6 embryonic stem cells; founder lines were mated to generate homozygous *nr4a2*^*fl/fl*^ mice and neomycin cassettes were removed by intercrossing with C57BL/6 FLPe mice (Riken BRC, Tsukuba, Japan). CD4-specific cKO animals were obtained by mating with C57BL/6 *CD4-Cre* mice (Taconic, Germantown, NY, USA) and backcrossing with *nr4a2*^*fl/fl*^, to obtain C57BL/6 *Cre-CD4 nr4a2*^*fl/fl*^ (refs 47, 48). *eomes*^*fl/fl*^ mice, purchased from Jackson Laboratories, were crossed with *Cre-CD4* and *Cre-CD4/nr4a2*^*fl/fl*^ mice, to generate Eomes cKO and NR4A2/Eomes DKO. For EAE induction, mice were injected subcutaneously with 100 μg MOG_35–55_ peptide (synthesized by Toray Research Center, Chūō-ku, Tokyo, Japan) and 1 mg heat-killed *Mycobacterium tuberculosis* H37RA emulsified in complete Freund’s adjuvant (Difco, KS, USA). One hundred nanograms of Pertussis toxin (List Biological Laboratories, CA, USA) was injected intraperitoneally on days 0 and 2 after immunization. EAE was clinically scored daily (0, no clinical signs; 0.5, tail weakness; 1, partial tail paralysis; 1.5, severe tail paralysis; 2, flaccid tail; 2.5, flaccid tail and hind limb weakness; 3, partial hind limb paralysis; 4, total hind limb paralysis; 5, hind and fore leg paralysis). For transfer EAE, sorted CNS T cells were activated *in vitro* with anti-CD3/antiCD28 for 24 h before intravenous (i.v.) transfer. For *invivo* systemic siRNA treatment, mice received i.v. 40 μM Eomes-specific siRNA (sequence 5′- ggctcttatttctactcatUU -3′; synthesized by Koken, Bunkyō-ku, Tokyo, Japan) or negative control siRNA stabilized with an AteloGene collagen systemic kit (Koken). To block PAR-1 receptors, mice were treated with 5 mg kg^−1^ intraperitoneal FR171113 (Sigma-Aldrich, Tokyo, Japan) in 0.5% carboxymethyl cellulose (Sigma-Aldrich); control vehicle was carboxymethyl cellulose alone.

### Cell isolation and purification

Single-cell splenocyte and lymph node cell suspensions were generated by mechanical disruption of tissues. CNS-infiltrating lymphocytes were isolated from the spinal cords and brains as previously described^49^,^50^. Briefly, the tissue was cut into small pieces and digested for 40 min at 37 °C in RPMI 1640 media (Invitrogen, Tokyo, Japan) supplemented with 1.4 mg ml^−1^ collagenase H and 100 μg ml^−1^ DNase I (Roche Diagnostics, Tokyo, Japan). Resulting tissue homogenates were forced through a 70-μm cell strainer and leukocytes were enriched using a discontinuous 37%/80% Percoll density gradient centrifugation (GE Healthcare Life Sciences, Tokyo, Japan). For transfer, CNS-infiltrating T cells were stained and purified using FACS sorting with a FACS ARIA II (BD Cytometry Systems, NJ, USA), before overnight restimulation with anti-CD3 and anti-CD28 antibodies (2C11 Hybridoma and Biolegend, Bunkyō-ku, Tokyo, Japan), and transferred by i.v. injection into immunized recipient mice.

### Microarray analysis

Expression microarrays were carried out on sorted CNS T cells using GeneChip Mouse Genome 430 2.0 Arrays prepared using a GeneChip 3′ IVT express Kit and a GeneChip Hybridization, Wash, and Stain Kit (all from Affymetrix, Tokyo, Japan) according to manufacturer’s instructions. Arrays were washed using a GeneChip Fluidics Station 450 and scanned using a GeneChip Scanner 3000 7G. Array data were compiled using Affymetrix GCOS software and analysed with GeneSpring GX software (Agilent Technologies, Santa Clara, CA).

### Human samples

The study protocol was approved by the Ethics Committee of National Center of Neurology and Psychiatry and written informed consent was obtained from all subjects. Blood samples from patients or healthy volunteers were collected in heparinized tubes and PBMCs were separated by underlaying a Ficoll gradient (Ficoll Paque Plus, GE Healthcare) and cetrifugation at 400*g* for 30 min. CSF cells were collected from freshly withdrawn CSF from SPMS volunteers by centrifugation at 600g.

### Assessment of cell function

Culture media used was DMEM supplemented with 10% FCS, 2 mM L-glutamine, 100 U ml^−1^ of penicillin–streptomycin and 50 μM 2-mercaptoethanol (all from Invitrogen). Where indicated, cells were activated with 2 μg ml^−1^ immobilized anti-CD3 mAb (2C-11) and 1 μg ml^−1^ anti-CD28 mAb (Biolegend). Cytokine concentrations in supernatants after stimulation were measured by FlowCytomix cytometric bead array (eBioscience, San Diego, CA, USA) according to the manufacturer’s instructions. For intracellular staining, cells were restimulated with 5 ng ml^−1^ PMA and 500 ng ml^−1^ ionomycin (both Sigma-Aldrich) in the presence of Golgi Stop (BD Biosciences) for 5 h, before surface staining and fixing/intracellular staining using a eBioscience Foxp3 staining kit according to manufacturer’s instructions. For CD107a antibody membrane trapping, cells were stimulated with anti-CD3/anti-CD28 antibodies in the presence of an anti-CD107a-flourochrome-conjugated antibody and GolgiPlug (BD Biosciences). Flow cytometry data were aquired using a FACS Canto II or FACS ARIA II (BD Cytometry Systems) with FACS DIVA software. Antibodies for flow cytometry were sourced from BD Biosciences, BioLegend or eBioscience as listed in Supplementary Tables 2 and 3. For quantification of messenger RNA transcripts, total RNA was extracted from cell populations using an RNeasy Mini Kit or FastLane kit (Qiagen, Chūō-ku, Tokyo, Japan) according to the manufacturer’s instructions and complementary DNA was then generated using a first-strand cDNA Kit (Takara, Otsu, Shiga, Japan). Quantified real-time PCR using either a Light Cycler-FastStart DNA Master SYBR Green I kit with a LightCycler instrument (Roche Diagnostics) or a Power SYBR green master mix with an ABI 7300 real-time PCR instrument (Applied Biosystems, Warrington, UK) was performed using commercial primers (Quantitech primers, Qiagen) and gene expression values were normalized to the expression of the *GAPDH*, *HPRT1* or *β2M* housekeeping genes.

## Additional information

**Accession codes:** Accession number for the microarray GSE72301.

**How to cite this article:** Raveney, B. J. E. *et al*. Eomesodermin-expressing T-helper cells are essential for chronic neuroinflammation. *Nat. Commun.* 6:8437 doi: 10.1038/ncomms9437 (2015).

## Supplementary Material

Supplementary Information Supplementary Figures 1-10 and Supplementary Tables 1-3
