# Supplementary material for: Eomesodermin-expressing T-helper cells are essential for chronic neuroinflammation
Source: Nat Commun. 2015 Oct 5;6:8437. doi: 10.1038/ncomms9437 (PMC4600741; doi:10.1038/ncomms9437)
Supplement: Supplementary Information — Supplementary Figures 1-10 and Supplementary Tables 1-3 [file ncomms9437-s1.pdf]

Supplementary Figure 1

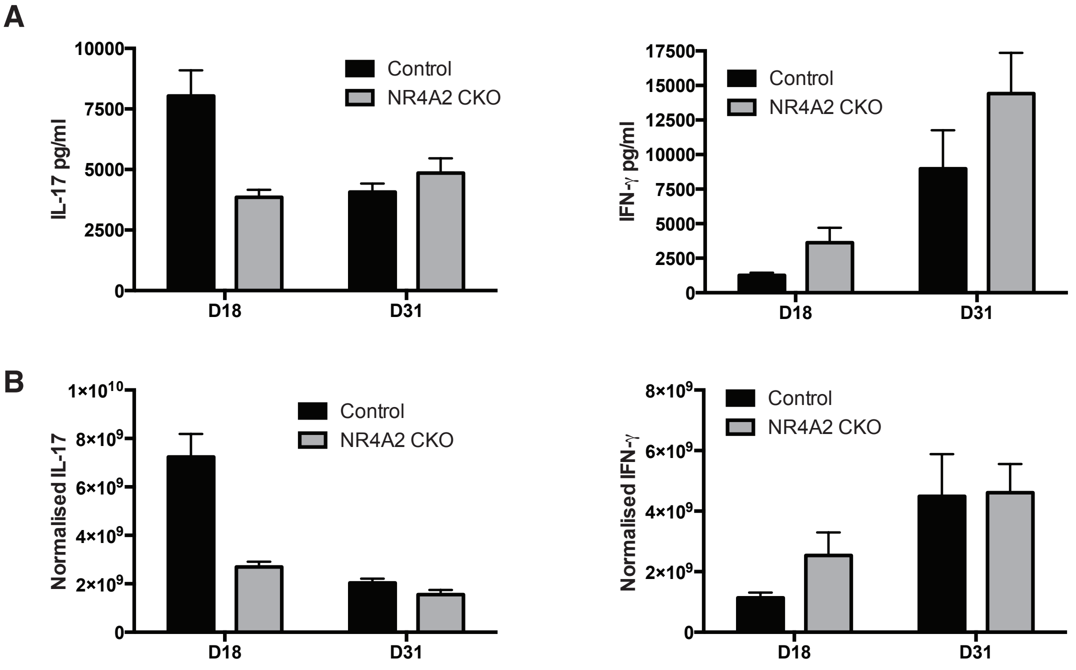

Supplementary Figure 1. Characterisation of CNS cell infiltrate during EAE in NR4A2 cKO mice

A and B: Ex vivo cytokine production from CNS CD4$^+$ T cells

CNS CD4$^+$ T cells were purified by cell sorter from control or NR4A2 cKO mice at early (Day 18) or late disease phase (Day 31). Equal numbers of cells were restimulated with anti-CD3/CD28. **A,** IL-17 and IFN-γ levels in supernatants after 96 hours. **B,** IL-17 and IFN-γ levels after 96 hours normalized to the number of infiltrating cells per CNS measured in arbitrary units.

Supplementary Figure 2

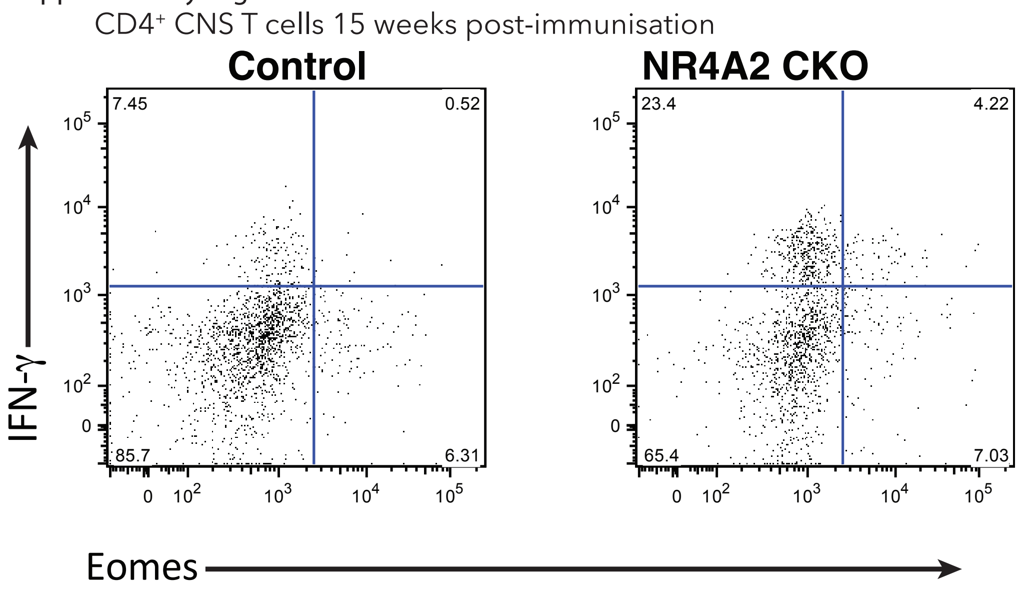

Supplementary Figure 2. Activated T cells are long-lived in the CNS tissue during chronic EAE

EAE was actively induced with MOG_{35-55} in WT mice (Control) and NR4A2 cKO mice. After 15 weeks, CNS-infiltrating cells were isolated and restimulated with PMA/ionomycin for 5 hours in the presence of golgiplug. Cells were then stained intracellularly for eomes and IFN-γ. Data are representative of 3 similar experiments.

Supplementary Figure 3

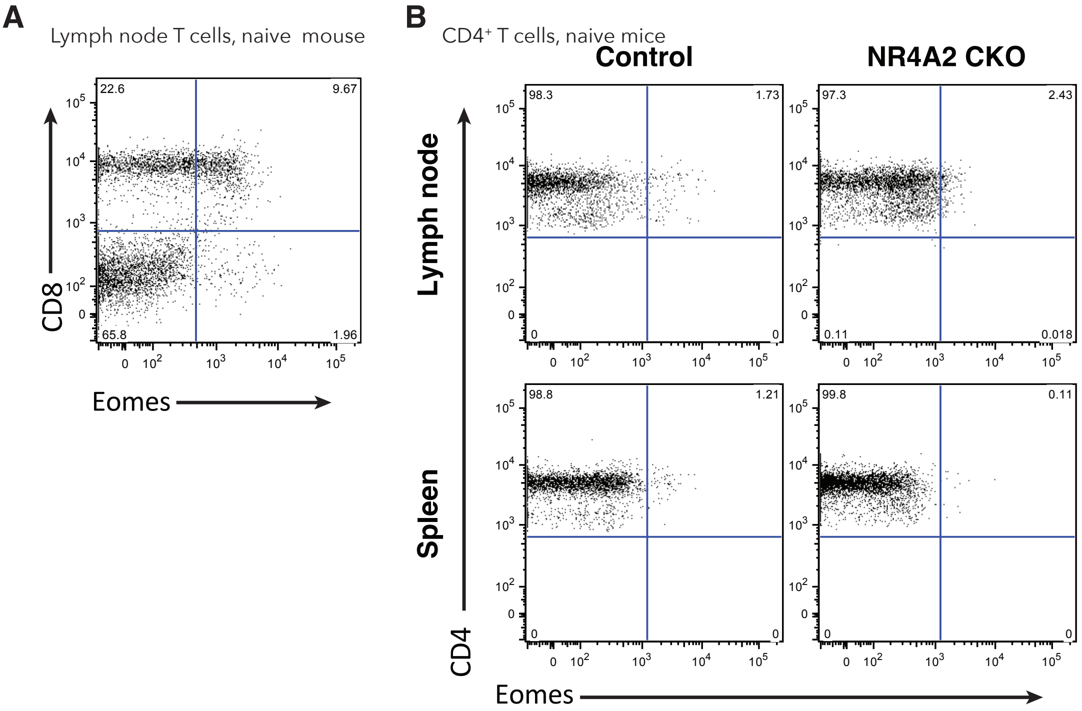

Supplementary Figure 3. Eomes expression in CD8^{+} and CD4^{+} T cells in the secondary lymphoid tissue

Isolated T cells from the secondary lymphoid tissue of unmanipulated naïve mice were stained for intracellular expression of Eomes and analyzed by flow cytometry. **A**, Eomes expression by CD8^{+} T cells in lymph nodes from naive WT mice. **B**, Eomes expression by CD4^{+} TcRβ^{+} cells from lymph nodes or spleen of control or NR4A2 cKO naive mice.

Supplementary Figure 4

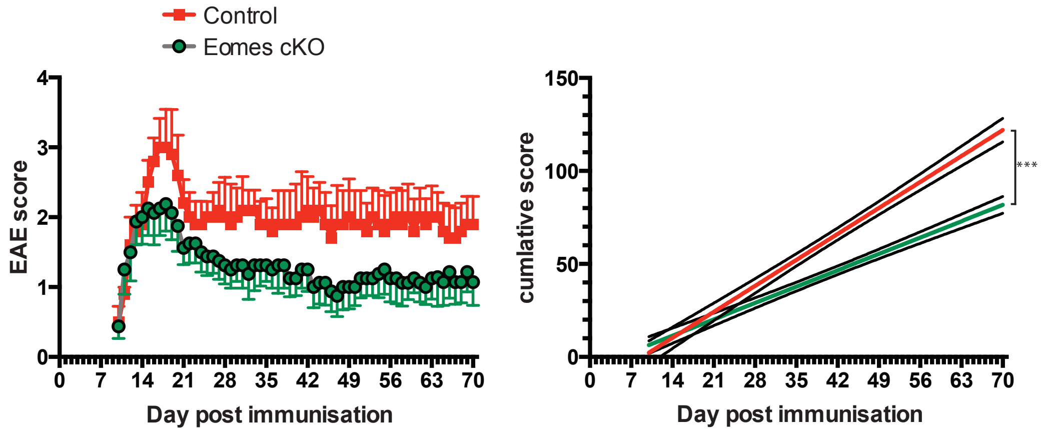

Supplementary Figure 4. Late stage clinical EAE is reduced in Eomes cKO mice Plots show disease scoring for control mice (red) verses mice lacking Eomes (Eomes cKO, green). Left panel shows daily clinical scores with error bars (SEM), whereas right plot shows cumulative disease burden with black lines showing 95% confidence intervals ***p<0.001 tested by linear regression analysis. Plots are representative of 2 independent experiments.

Supplementary Figure 5

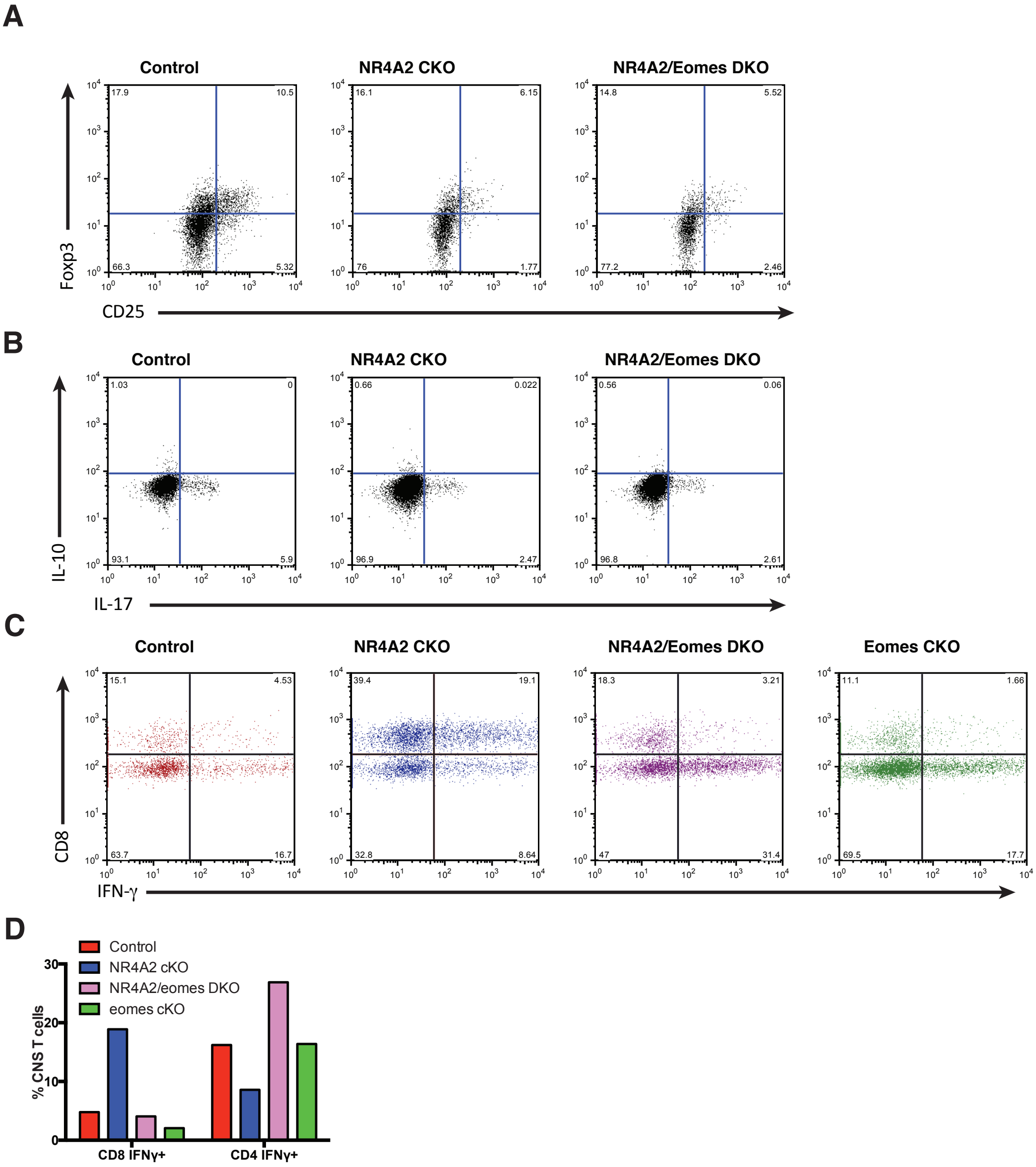

Supplementary Figure 5. CNS T cell phenotypes in late stage EAE

A, CD25 and intracellular Foxp3 staining for $CD4^+$ CNS T cells from the indicated genotypes on day 30 post immunisation. **B**, IL-17 and IL-10 staining for $CD4^+$ CNS T cells restimulated with PMA/ionomycin for 5.5hours in the presence of golgiplug at day 30 post EAE induction. **C**, CD8α and intacellular IFN-γ staining of CNS T cells restimulated with PMA/ionomycin for 5.5hours in the presence of golgiplug on day 27 post EAE induction. **D**, summary percentages of IFN-γ-producing cells of CNS T cell populations. Data are from 1 of 2 similar experiments of pools of 3 mice from each genotype.

Supplementary Figure 6

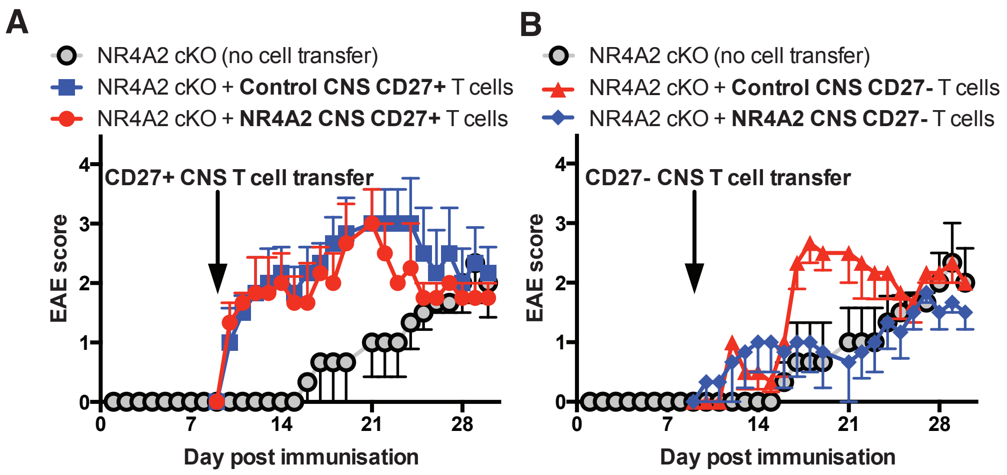

Supplementary Figure 6. Late stage CD27^{+} CNS T cells from NR4A2 cKO mice are pathogenic

Clinical EAE scores of $MOG_{35\text{-}55}$-immunised NR4A2 cKO mice receiving a transfer of 3 x 10^4 CNS T cells restimulated with anti-CD3/anti-CD28 antibodies for 24 hours from donor D28 immunised control mice (red squares) or NR4A2 cKO mice (blue circles) compared with NR4A2 cKO that received no transferred cells (grey circles). **A:** transfer of CD27^{+} T cells; **B:** transfer of CD27^{-} T cells. Plots are representative of experiments with 3 recipient mice per group.

Supplementary Figure 7

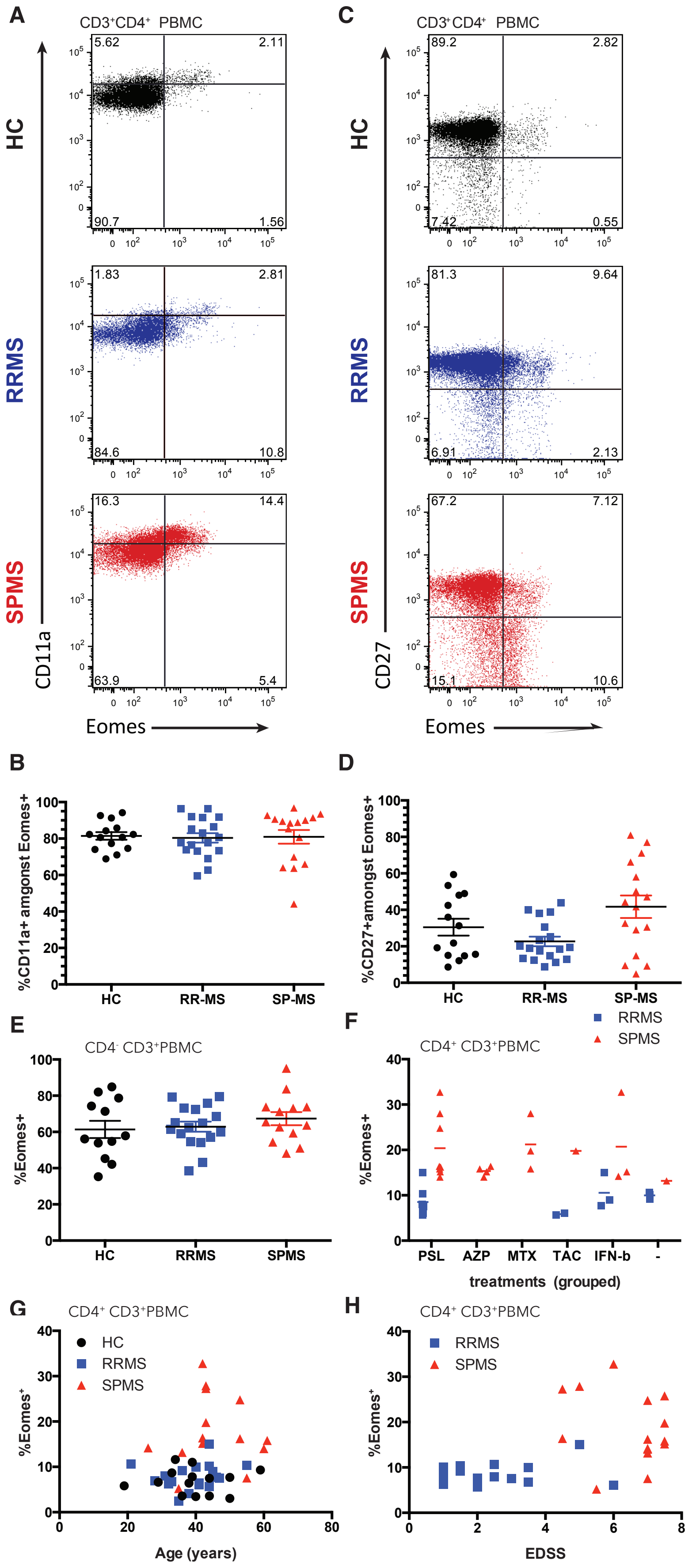

Supplementary Figure 7. Eomes expression in PBMC populations

Multicolor flow cytometric analysis for $Eomes^{+}CD4^{+}$T cells from healthy controls (HC), RRMS patients, or SPMS patients. **A**, Expression of Eomes versus CD11a in $CD3^{+}CD4^{+}$ PBMC and percentage CD11a co-expression with Eomes for each clinical group is summarized in **B**. **C,** Expression of Eomes versus CD27 in $CD3^{+}CD4^{+}$ PBMC and percentages of $CD27^{+}$ cells amongst $Eomes^{+}CD3^{+}CD4^{+}$ PBMC is summarized in **D**. **E**, Percentages of $Eomes^{+}$ T cells from the $CD4^{-}$ fraction of PBMC (% eomes+). **F,** Comparison for $Eomes^{+}$ T cells % amongst $CD4^{+}CD3^{+}$ PBMC (% eomes+) in RRMS or SPMS patients receiving different drug treatments. PSL=prednisolone, AZP=azathioprine, MTX=methotrexate, TAC=tacrolimus, IFN-b=interferon-β. **G**, Subject age and $Eomes^{+}$ T cells % amongst $CD4^{+}CD3^{+}$ PBMC (% eomes+) in HC, RRMS, and SPMS. **H,** Disability score, EDSS (Expanded disability status scale) and % eomes+. Data are derived from patient group detailed in supplementary table 3.

Regression analysis

Age: HC, r^2= 1.222e-005; RRMS, r^2= 0.01495;SPMS, r^2= 0.01977

EDSS: RRMS, r^2= 0.03786; SPMS, r^2= 0.1095

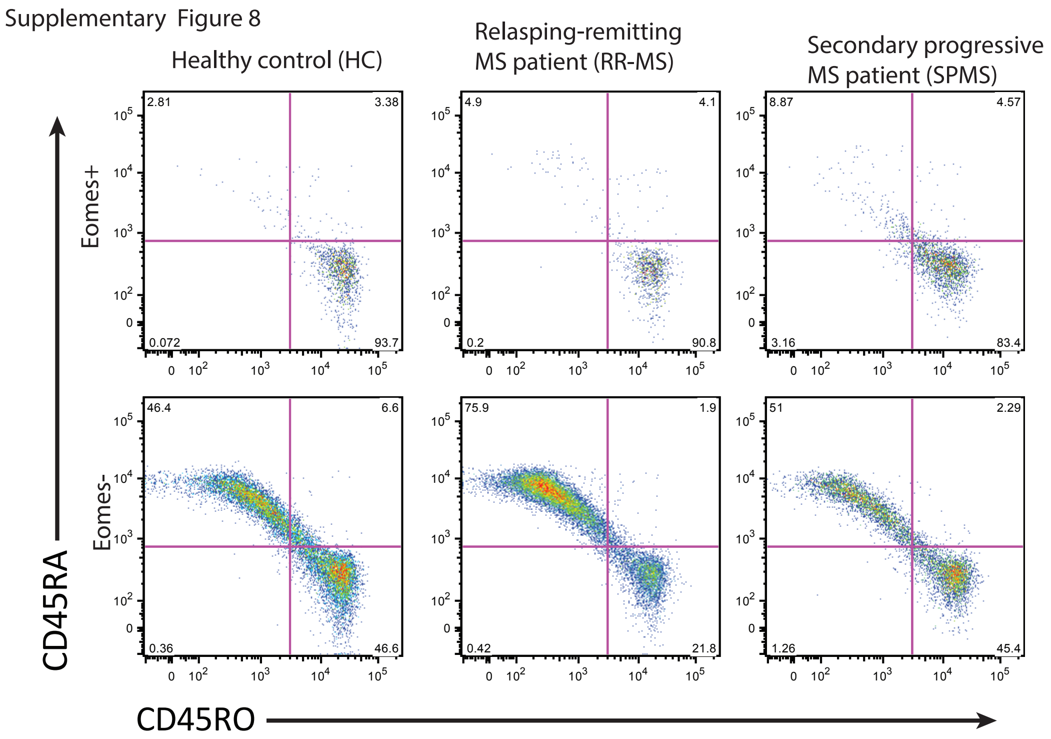

Supplementary Figure 8. Eomes expression in human CD4$^+$ T cells is associated with an activated CD45RO$^+$CD45RA$^-$ phenotype

CD45RO and CD45RA expression by Eomes$^+$ and Eomes$^-$ subsets of CD4$^+$CD3$^+$ PBMC from a representative subject from each patient/control group.

Supplementary Figure 9

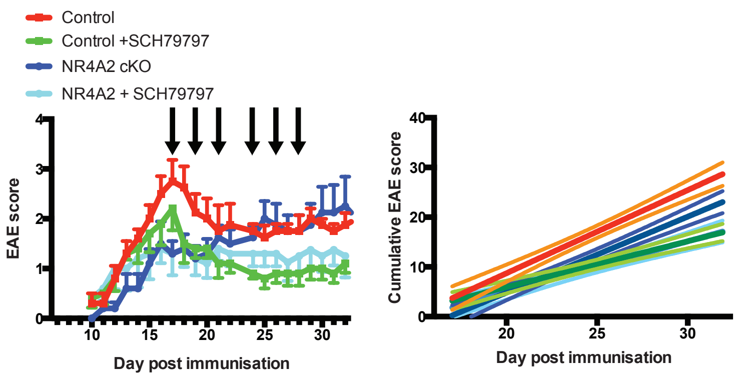

Supplementary Figure 9. PAR-1 specific antagonist reduces late/chronic EAE

Groups of control or NR4A2 cKO mice were immunized with MOG_{35-55} to induce EAE. One group of each genotype was treated on alternate days from day 17 post EAE induction by *i.p.* injection of the PAR-1-antagonist SCH79797 or vehicle (CMC). Left panel shows clinical EAE scores. Error bars represent SEM. Right plot shows cumulative disease, dashed lines show 95% confidence intervals, $^{**}p<0.01$ tested by linear regression analysis. $n \geq 5$ and data are representative of 2 independent experiments.

Supplementary Figure 10

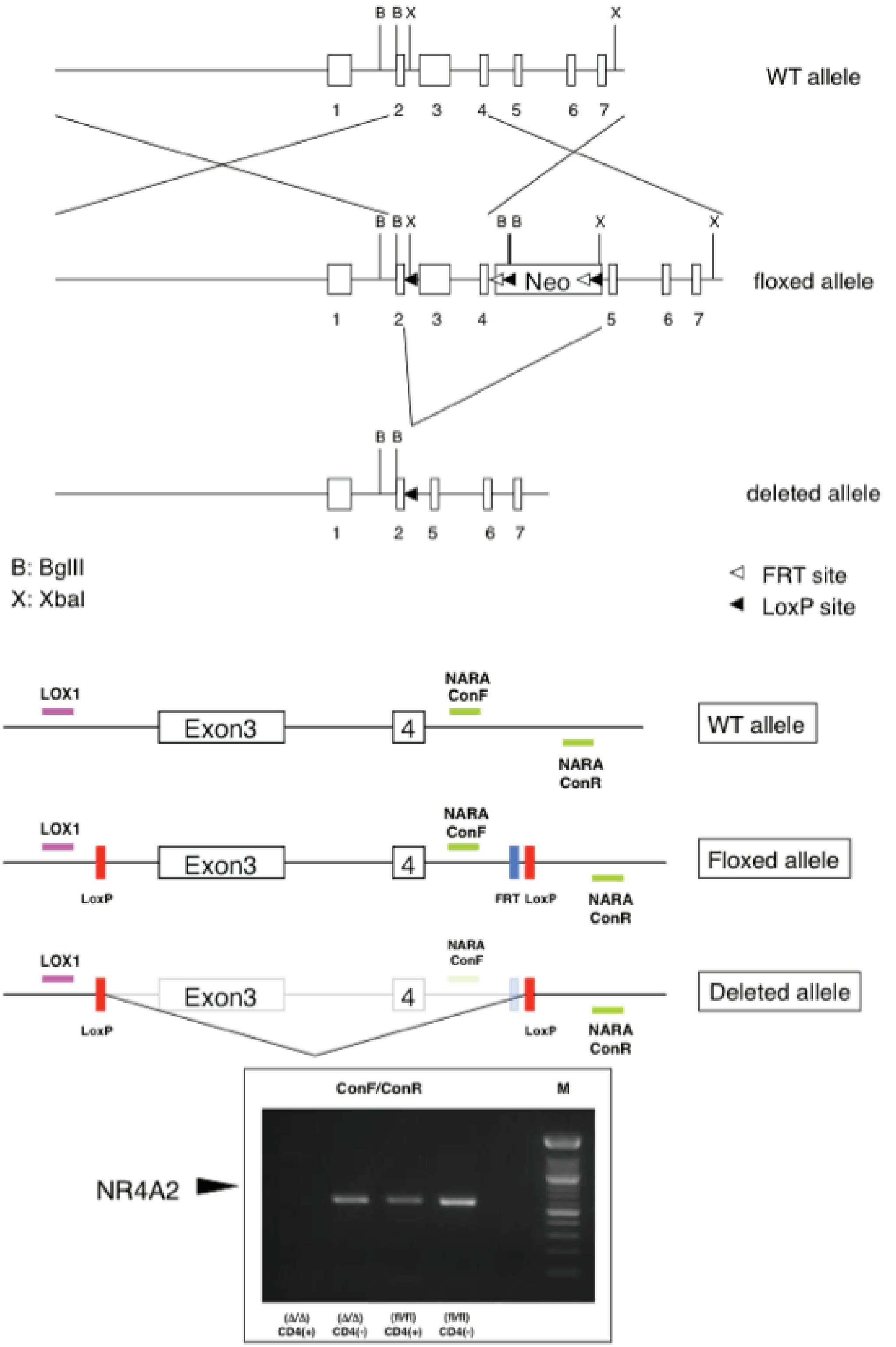

Supplementary Figure 10

Schematic diagram of the generation of NR4A2$^{fl/fl}$ mice

	HC	RRMS	SPMS
Number	14	17	16
Sex	8F 6M	11F 6M	10F 6M
Median age	39	40	43
Mean age (SEM)	39.57±2.63	38.65±1.94	46.33±2.69
Age range	19-59	21-60	26-61
Age s.d.	9.84	8.22	10.42

Supplementary Table 1

Table indicating the demographics of healthy control and MS patient groups relating to data in Fig. 5. HC, healthy control; RRMS, relapsing remitting MS; SPMS , secondary progressive MS.

Antigen	Clone	Supplier
CD16/32	93	Biolegend
CD3e	145-2C11	Biolegend
CD4	GK1.5	Biolegend
CD11a	H155-78	Biolegend
CD11b	M1/70	Biolegend
CD27	LG-7F9	eBioscience
CD45	30-F11	Biolegend
CD45	30-F11	eBioscience
TcRβ	H57-597	Biolegend
F4/80	BM8	Biolegend
eomes	Dan11Mag	eBioscience
IL-17	TC11-18H10.1	Biolegend
IL-17	eBio17B7	eBioscience
IFN-γ	XMG1.2	Biolegend
RORγt	B2D	eBioscience
Foxp3	FJK-16s	Biolegend

Supplementary Table 2
Anti-mouse antibodies used in this study

Antigen	Clone	Supplier
CD3	OKT3	Biolegend
CD4	OKT4	Biolegend
CD11a	HI111	Biolegend
CD27	0323	eBioscience
CD45RA	HI100	Biolegend
CD45RO	UCHL1	Biolegend
CD107a	H4A3	Biolegend
IL-17	BL168	Biolegend
IFN-γ	45.B3	Biolegend
Perforin1	dG9	Biolegend
Granzyme B	GB11	Biolegend
Eomes	WD1928	eBioscience

Supplementary Table 3
Anti-human antibodies used in this study
